# I GOT THIS! SUCCESSFUL AGING THROUGH THE MAINTENANCE OF AUTONOMY

**DOI:** 10.1093/geroni/igz038.2638

**Published:** 2019-11-08

**Authors:** Alexandria G Nuccio, Ashely M Stripling

**Affiliations:** 1 Nova Southeastern University, Davie, Florida, United States; 2 Nova Southeastern University, Fort Lauderdale, United States

## Abstract

As America ages, an increased interest has been placed on understanding the development and maintenance of autonomy in later life. This is of particular importance given the impact of autonomy on vitality, well-being, and mood within older adults (Warner et al., 2011). However, the research examining which aspects of autonomy directly impact successful aging remains underexplored. As such, the current study utilizes the Functional Autonomy Measurement System (SMAF) to better understand which facets of autonomy promote life satisfaction in older adults. Data included assessments of the Functional Autonomy Measurement System and data was derived from the Survey of Midlife in the US Database (MIDUS 3). Participants were primarily White/Caucasian (88.7%) and female (54.0%); with a mean age of 63.64 years (SD=11.35). A series of hierarchical multiple linear regressions revealed that higher levels of Mental Functions predicted increased life satisfaction scores in models adjusted for age, sex, race, marital status, and education (F=54.152,p<0.001) and that higher levels of Communication (F=37.553,p<0.001), Activities of Daily Living (F=33.495,p<0.001), Mobility (F=37.311,p<0.001), and Instrumental Activities of Daily Living (F=8.630,p<0.001) also predicted increases in life satisfaction scores but to a lesser extent. The present findings suggest that higher levels of autonomy, both physically and mentally, result in higher satisfaction with life; with perceptions of cognitive independence producing the highest levels. Implications of the current findings include increased understanding of the multifaceted nature of autonomy, and provision of recommendations which could increase autonomous behavior in later life, thus increase life satisfaction and promote successful aging.

